# A Case Report of Neuronal Intranuclear Inclusion Disease Presenting With Recurrent Migraine-Like Attacks and Cerebral Edema: A Mimicker of MELAS

**DOI:** 10.3389/fneur.2022.837844

**Published:** 2022-03-01

**Authors:** Fei Xie, Xingyue Hu, Peng Liu, Dan Zhang

**Affiliations:** ^1^Department of Neurology, School of Medicine, Sir Run Run Shaw Hospital, Zhejiang University, Hangzhou, China; ^2^Department of Neurology, School of Medicine, The Second Affiliated Hospital, Zhejiang University, Hangzhou, China

**Keywords:** neuronal intranuclear inclusion disease, migraine, MELAS, *NOTCH2NLC*, cerebral edema

## Abstract

**Background:**

Neuronal intranuclear inclusion disease (NIID) is a progressive neurodegenerative disease associated with the GGC repeats in the 5'-untranslated region (5'UTR) of *NOTCH2NLC*. NIID exhibits a wide range of clinical manifestations. However, patients presenting with recurrent migraine-like attacks and cerebral edema have only rarely been reported.

**Case Presentation:**

A Chinese female suffered probable migraines with aura for 10 years. At age of 51, aggravating migraine-like attacks co-occurred with a sudden encephalopathy-like episode. Brain MRI showed right cerebral edema and cortical enhancement. Serum lactic acid level was elevated at rest and significantly increased after a simplified serum lactic acid exercise test. The initial diagnosis was MELAS, while NIID was suspected after faint DWI high-intensity signals in the corticomedullary junction was retrospectively recognized. Mitochondrial genome sequencing and gene panel analysis of nuclear genes related to mitochondrial diseases failed to find any causative variants. Repeat-primed PCR and fluorescence amplicon length PCR of *NOTCH2NLC* gene identified an abnormal expansion of 118 GGC repeats in the 5'UTR of *NOTCH2NLC* gene.

**Conclusion:**

NIID should be taken into account for differential diagnosis of migraines and MELAS-like episodes. Besides the corticomedullary hyperintensity on DWI, cortical enhancement in contrast-enhanced brain MRI may also be a brain image marker for the differential diagnosis between MELAS and NIID with MELAS-like episodes.

## Introduction

Neuronal intranuclear inclusion disease (NIID) is a progressive neurodegenerative disease characterized by the presence of eosinophilic hyaline intranuclear inclusions in the central and peripheral nervous systems and various organs (1). Recently, trinucleotide GGC repeat expansion in the 5'-untranslated region (5'UTR) of the *NOTCH2NLC* gene has been confirmed as the cause of familial and sporadic cases of NIID (2–4). NIID exhibits a wide range of clinical manifestations, including dementia, sensory disturbances, autonomic nerve dysfunction, paroxysmal disturbances in consciousness, muscle weakness, cerebellar ataxia, parkinsonism, as well as other neurological and psychiatric symptoms (1). Encephalopathy-like episodes can manifest in some patients with NIID (1, 5). However, a mitochondrial encephalomyopathy, lactic acidosis, and stroke-like (MELAS)-like episode in long-standing history of migraine with aura has only been rarely reported in some NIID cases. Therefore, we reported a NIID patient, who was clinically diagnosed as migraine with aura for 10 years, with an encephalopathy-like episode which mimicked MELAS.

## Case Presenting

A 51-year-old female presented with recurrent headaches for 10 years. At the age of 41, she had probable migraines with aura. The headache attacks usually preceded by a paroxysmal blurred vision, which looked like a snowflake-like flashing dots in front of her eyes. These vision symptoms usually lasted for about 15 min, followed by numbness on the left fingers and the lip, which lasted for about 5 min. A severe throbbing headache in the right frontotemporal region occurred next, which sometimes accompanied by nausea and vomiting. Photophobia and phonophobia during attacks were also reported. The pain was often severe enough to hamper daily activities and may last from hours to half a day when painkillers were given. Initially, she had 3 to 4 attacks each year. Four years ago, she started to take flunarizine to prevent migraine when headaches increased. Two month before admission, she stopped taking flunarizine, and the headaches occurred twice a month.

**Figure 1:**
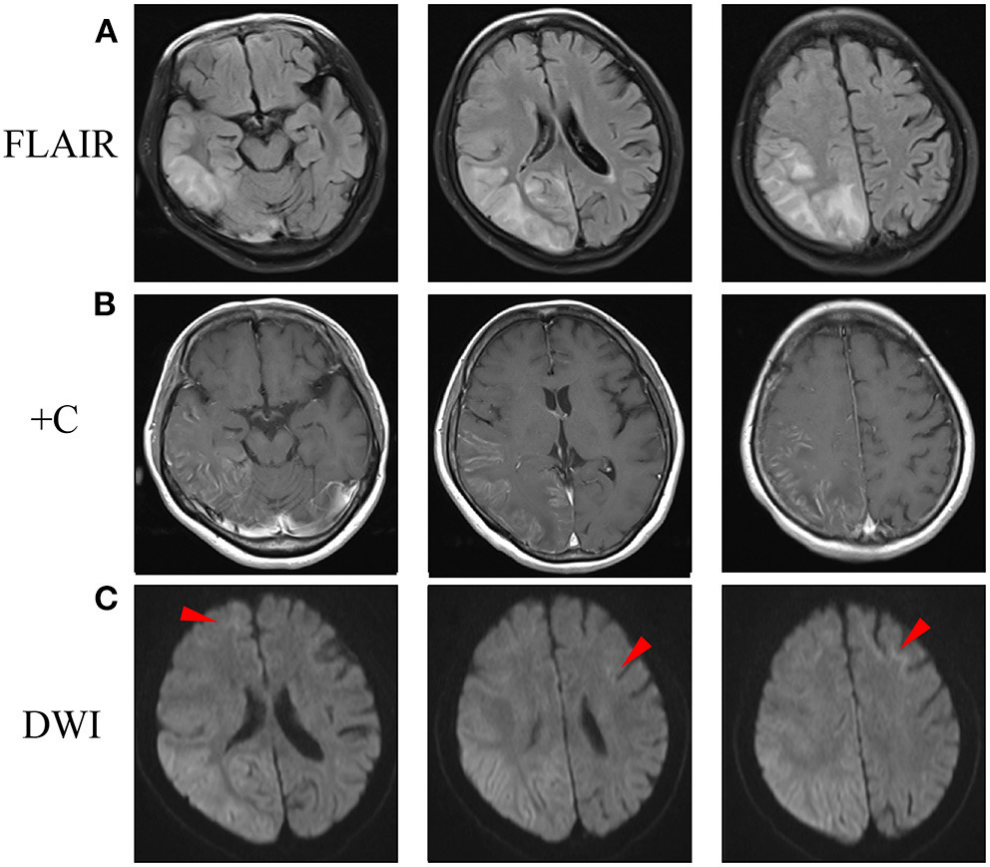
Brain MRI scan after admission. **(A)** FLAIR images showed abnormal hyperintensity along the cortex and partial subcortical area in the right occipito-parieto-temporal lobes and a part of frontal lobe. **(B)** Post-contrast MRI showed enhanced lesions along the surface of the cortex. **(C)** DWI showed hyperintensity in the right cerebral and corticomedullary junction of anterior frontal lobes (arrowheads). DWI, diffusion-weighted image; FLAIR, fluid-attenuated inversion recovery.

At the age of 51, half a month before admission, she experienced a headache after visual and sensory auras. However, unlike her previous attacks, the headache and numbness persisted for 10 days, so she sought for medical help. Fever or consciousness disturbance was not reported. Brain magnetic resonance imaging (MRI) showed abnormal hyperintensities on diffusion-weighted images (DWI), T_2_-weighted images and fluid-attenuated inversion recovery (FLAIR) images along the cortex and partial subcortical area in the right occipito-parieto-temporal lobes and part of frontal lobe, with gadolinium enhancement selectively spreading along the surface of the cortex (Figures 1A,B). She was admitted for further evaluation. She was alert and well-oriented. Neurological examination revealed absence of tendon reflexes, with normal cranial nerves, muscle strength, sensation, and Babinski exams. Mini-Mental Status Examination scored 28. Cranial magnetic resonance angiography was normal. Cerebrospinal fluid analyses showed only mild protein elevation (0.61, normal range 0.15–0.45 g/L), without pleocytosis. Cerebrospinal fluid autoimmune encephalitis antibodies, including anti-NMDAR, anti-AMPAR1, anti-AMPAR2, anti-LGI1, anti-CASPR2, and anti-GABAR were all negative. Serum paraneoplastic antibodies, including anti-Hu, anti-Yo, anti-Ri, anti-CV2, anti-Ma2, and anti-Amphiphysin, were all negative. Serum lactic acid level was elevated at rest and significantly increased after simplified serum lactic acid exercise test. Serum lactic acid level at rest, immediately after exercise, and 10 min after exercise was 2.20, 9.30, 8.20, respectively (normal range 0.9–1.7 mmol/L). Besides, she complained of difficulty in urination for half a year. Ultrasound revealed urinary retention. Urodynamic evaluations revealed a bladder capacity of 140 ml, the pressure of detrusor higher than 40 cmH_2_O when filling more than 130 ml saline and an inability to urinate during voiding stage. She complained neither light-headedness nor syncope while standing up. Electromyogram and nerve conduction studies and other autonomic function tests were not performed. Initially, with cortical involvement and positive lactic acid test, MELAS was suspected. Coenzyme Q10 and riboflavin were given. However, NIID was suspected as faint DWI high-intensity signals in the corticomedullary junction was recognized when we re-evaluated her brain MRI image (Figure 1C).

**Figure 2:**
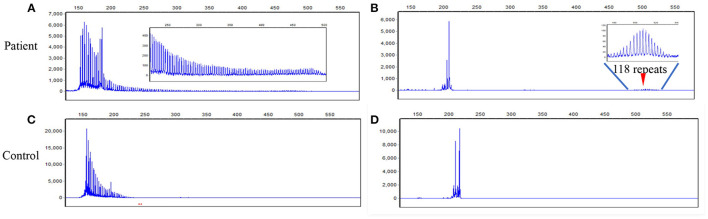
RP-PCR and fluorescence AL-PCR in the patient and a negative control. **(A)** RP-PCR showed a characteristic saw-tooth pattern in the patient. **(B)** Fluorescence AL-PCR showed expanded allele had an unusual peak at around 507 bp (arrowheads) in the patient. The GGC repeat number of expanded allele was calculated according to the length of the highest fluorescent peak in expanded alleles [GGC repeat number = (507–154)/3]. **(C)** RP-PCR showed only single peak wave without a saw-tooth pattern in a negative control. **(D)** Fluorescence AL-PCR showed no repeat expansion in a negative control. RP-PCR, repeat-primed PCR; AL-PCR, amplicon length PCR.

DNA test was performed with written informed consent. DNA was extracted from peripheral leukocytes using standard methods. A next-generation sequencing-based whole mitochondrial genome analysis and a comprehensive gene panel analysis containing nuclear genes related to mitochondrial diseases failed to find any causative variants. Repeat-primed PCR (RP-PCR) and fluorescence amplicon length PCR (AL-PCR) was performed to screen the GGC expansion in the 5'UTR of the *NOTCH2NLC* gene (2). The long saw-tooth curves in RP-PCR indicated that GGC expansion is positive (Figures 2A,C). Fluorescence AL-PCR revealed that the number of GGC repeat expansion was 118 (Figures 2B,D). Her mother and one sister had no history of chronic headache or other neurological disease. However, her father suffered from brain atrophy for more than 10 years, with his last 2 years of life bed-ridden. Unfortunately, neuroimaging and details of her father's disease was not available. She had a healthy daughter in her twenties. No DNAs of other family members were available. Skin biopsy was refused.

**Figure 3:**
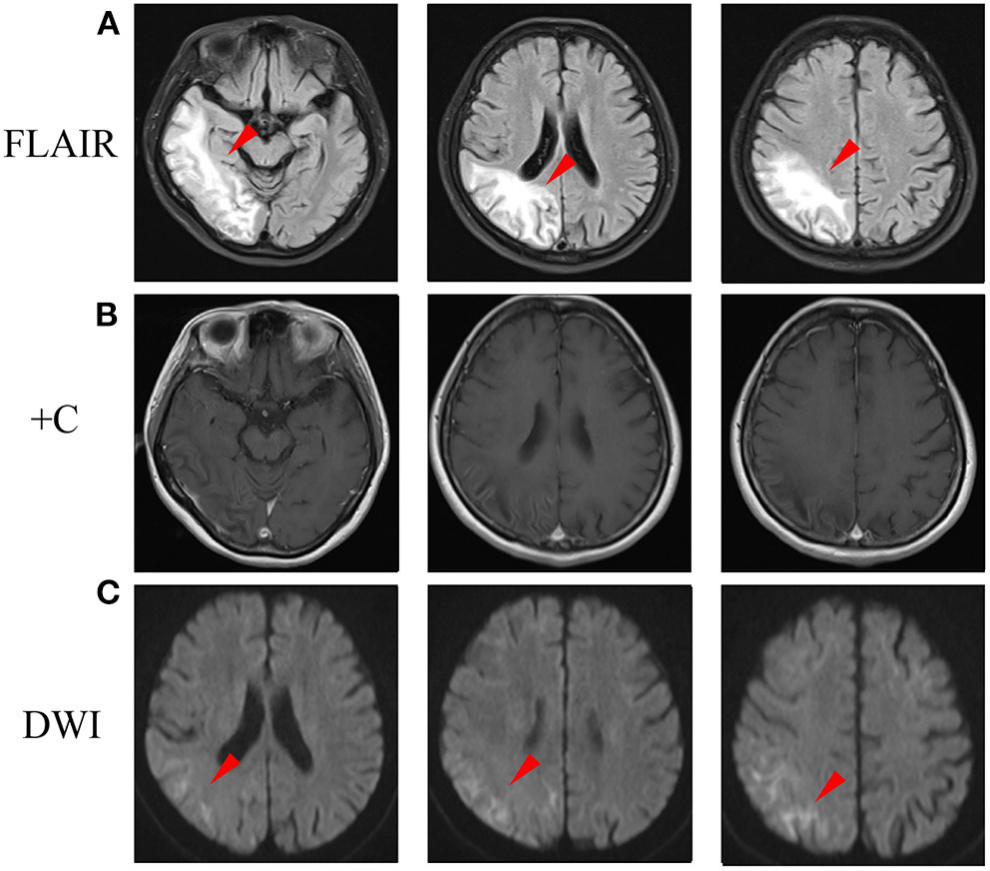
A follow-up brain MRI performed 3 months later after disease onset. **(A)** FLAIR images showed abnormal hyperintensity was improved in cortex, while progressed in the subcortical area (arrowheads). **(B)** Post-contrast MRI showed cortical enhancement was reduced. **(C)** DWI showed hyperintensity in the corticomedullary junction progressed (arrowheads). DWI, diffusion-weighted image; FLAIR, fluid-attenuated inversion recovery.

During the follow-up, she was continued with coenzyme Q10 and riboflavin. Fatigue and poor appetite was complained, and no headache was reported. A follow-up brain MRI performed 3 months later after disease onset revealed the cortical enhancement was improved (Figure 3B), while DWI high-intensity lesions in the corticomedullary junction and FLAIR high signals in the subcortical area progressed (Figures 3A,C).

## Discussion

In this study, we report a patient with NIID presenting with a MELAS-like episode in long-standing history of migraine with aura. Our patient present with two rare situations occurred in NIID patients. First, migraine with aura and NIID occur concurrently in our patient, a condition that was only reported in a few patients with NIID. Our case gives further evidence that the two diseases could co-relate with each other. Second, extensive cerebral edema, with the cerebral cortex mainly involved, and elevated serum lactic acid level, strongly suggest a MELAS-like episode. This is a very uncommon presentation in NIID patients. Our case indicated again that differential diagnosis should be emphasized between adult-onset NIID and MELAS.

Adult-onset NIID exhibit highly variable clinical manifestations and the typical phenotype was categorized into a dementia-dominant subtype and a weakness-dominant subtype (1). However, GGC repeat expansion in *NOTCH2NLC* is also associated with essential tremor, Alzheimer's disease, frontotemporal dementia, Parkinson's disease, multiple system atrophy, leukoencephalopathy, amyotrophic lateral sclerosis, and oculopharyngodistal myopathy type 3 (6). However, recurrent migraines and MELAS-like episodes are quite atypical manifestations, and only a few cases have been reported previously. Wang et al. described a juvenile-onset NIID patient in whom hemiplegic migraine-like headache and NIID occurred simultaneously (7). Liang et al. reported two NIID patients presenting with migraine with aura as the initial symptom, and the migraine attack co-occurred with encephalopathy-like episode subsequently (8). Zhao et al. reported a NIID patient suffered from long-term recurrent vestibular migraine-like attacks (9). Our patient had a similar disease course compared with that reported by Liang et al. (8). As migraine is a very common disease, whether the two disorders are two independent clinical conditions or co-relate with each other is still controversial. More cases and further researches are needed to establish their relationship. However, the evolution of clinical symptoms in these cases, from the earlier isolated migraine-like attack to the later migraine-like attack co-occurred with encephalopathy-like episode, suggest a coherent process of one disease at different stages (9). These cases remind us that migraine may present as the initial symptom of adult-onset NIID. Therefore, NIID should be considered as a possible differential diagnosis in migraine patients with atypical clinical symptoms and positive radiological findings.

Typical DWI high-intensity signals along the corticomedullary junction is a strong indicator for the suspected diagnosis of NIID. Additionally, DWI high signals on the corpus callosum and symmetrical T2 high signals of the cerebral white matter are also useful diagnostic clues. Cerebral edema not corresponding to the vascular distribution has only been reported in a few cases of NIID (5, 7, 8, 10). This could be easily misdiagnosed as MELAS when there was no abnormal corticomedullary hyperintensity on DWI (8, 10). Actually, our patient had been initially considered as MELAS and NIID was suspected as faint DWI high-intensity signals in the corticomedullary junction was retrospectively recognized. We summarized case reports of NIID patients with MELAS-like episode in Table 1. All the patients with contrast-enhanced MRI exhibited cortical enhancement, except one (7). Therefore, this may also be a brain imaging marker for the differential diagnosis between the two conditions. Besides the encephalopathy episode, peripheral neuropathy and cognitive impairment were frequently reported in NIID patients with MELAS-like episode (Table 1). However, cognitive impairment and peripheral neuropathy could also be manifestations of MELAS (11). Interestingly, pathologic mitochondrial abnormalities in the skeletal muscle has also been reported in a NIID patient (12). These results indicated again that mitochondrial dysfunction might potentially be associated with NIID patients with MELAS-like episode.

**Table 1:** The clinical and brain MRI features of NIID patients with MELAS-like episode.

**References**	**Patient no**.	**No. of GGC repeats**	**Gender** **/age**	**Headache**	**Episodic encephalo-** **pathy**	**Other clinical phenotype**	**Brain edema**	**Corticomedullary hyperintensity on DWI**	**Cortical enhancement in MRI**	**Treatment**
Our study	1	118	F/51	Migraine	+	PN?, AD	+	+	+	Coenzyme Q10, riboflavin
Wang et al. (7)	2	N. A.	M/20	HM	+	PN, seizures	+	+	–	ASM
Okubo et al. (5)	3	143	M/50	N. A.	+	PN, CI, Tremor	+	+	N. A.	N. A.
Ishihara et al. (10)	4	^*^	F/47	N. A.	+	PN	+	–	+	ASM, edaravone, taurine
Liang et al. (8)	5	115	F/56	Migraine	+	CI	+	–	+	Methylprednisolone, dehydration
	6	98	F/35	Migraine	+	CI	+	+	+	Methylprednisolone, dehydration
	7	123	M/56	Headache	+	CI, PN, AD	+	+	+	Methylprednisolone, dehydration
	8	110	F/61	Headache	+	CI, PN, AD, Tremor	+	+	+	Methylprednisolone, dehydration

*No., number; N.A., Not available; HM, hemiplegic migraine; PN, peripheral neuropathy; CI, cognitive impairment; AD, autonomic dysfunction; ASM, anti-seizure medications;*

* *(GGC)88(GGGA)1{(GGC)4(GGA)2}9(GGC)4(GGA)1(GGC)3(GGA)2(GGC)2*.

In conclusion, NIID should be considered in the differential diagnosis of migraine and MELAS-like episodes. Besides the corticomedullary hyperintensity on DWI, cortical enhancement in contrast-enhanced MRI may also be a brain image marker for the differential diagnosis between MELAS and NIID with MELAS-like episode.

## Data Availability Statement

The original contributions presented in the study are included in the article/supplementary material, further inquiries can be directed to the corresponding author/s.

## Ethics Statement

This study was approved by the Research Ethics Committees of the Sir Run Run Shaw Hospital, Zhejiang University, School of Medicine. The patients/participants provided their written informed consent to participate in this study. Written informed consent was obtained from the individual(s) for the publication of any potentially identifiable images or data included in this article.

## Author Contributions

FX designed the study and wrote the first draft of the manuscript. XH offered suggestions for the study and revised the manuscript. PL performed the genetic analysis and revised the manuscript. DZ organized the study, collected clinical data, and revised the manuscript. All authors reviewed and accepted the final manuscript.

## Conflict of Interest

The authors declare that the research was conducted in the absence of any commercial or financial relationships that could be construed as a potential conflict of interest.

## Publisher's Note

All claims expressed in this article are solely those of the authors and do not necessarily represent those of their affiliated organizations, or those of the publisher, the editors and the reviewers. Any product that may be evaluated in this article, or claim that may be made by its manufacturer, is not guaranteed or endorsed by the publisher.
